# Pigment Epithelium-Derived Factor (PEDF) as a Regulator of Wound Angiogenesis

**DOI:** 10.1038/s41598-018-29465-9

**Published:** 2018-07-24

**Authors:** Elizabeth R. Michalczyk, Lin Chen, David Fine, Yan Zhao, Emman Mascarinas, Paul J. Grippo, Luisa A. DiPietro

**Affiliations:** 1 Center for Wound Healing and Tissue Regeneration, College of Dentistry, University of Illinois at Chicago, Chicago, IL USA; 2 Division of Gastroenterology and Hepatology, Department of Medicine, College of Medicine, University of Illinois at Chicago, Chicago, IL USA

## Abstract

Although the inflammatory and proliferative phases of wound healing have been well described, much less is known about how healing resolves. During the resolution phase, pruning of the capillary bed and maturation of capillaries occurs and influences the final strength and fidelity of the wound. PEDF, an endogenous anti-angiogenic factor, is produced in wounds and may contribute to the removal of capillaries during wound resolution. This study utilized PEDF^−/−^ mice to examine how PEDF influences wound angiogenesis, particularly capillary density and permeability. The absence of PEDF led to transient changes in dermal wound closure and collagen content, but caused substantial changes in wound angiogenesis. Compared to wild type (WT) mice, wounds from PEDF^−/−^ mice exhibited a significant increase in capillaries during the proangiogenic phase of repair, and a delay in capillary pruning. Conversely, the addition of rPEDF caused a reduction in capillary density within skin wounds in WT mice. *In vitro* studies showed that PEDF inhibited migration and tube formation by dermal microvascular endothelial cells, and caused a decrease in the expression of VEGFR2, VCAM-1, and other surface receptors. The results demonstrate that loss of PEDF causes a distinctive wound healing phenotype that is characterized by increased angiogenesis and delayed resolution. The findings suggest that PEDF most likely acts through multiple mechanisms to regulate proper capillary refinement in wounds.

## Introduction

The repair of wounds is a complex biological process that requires the coordination of multiple cell types and their microenvironment^1^. One important characteristic of healing wounds in skin is a robust increase in capillary density, or angiogenesis^2,3^ Sprouting and branching of blood vessels results in an extensive yet immature network that eventually resolves by systematic pruning of vessels as healing proceeds^4^. The pruning of the vasculature is important to wound resolution, and occurs in tandem with remodeling of the newly formed extracellular matrix (ECM). In the early stages of wound healing, dermal fibroblasts fill the wound with primarily Collagen Type III, whereas the late stages are dominated by the production of Collagen Type I. As the wound resolves, the collagen ultrastructure becomes further refined as the collagen increases fibrillary size, undergoes cross-linking, and develops a basket weave orientation similar to normal skin^5^. During this period, capillaries regress, and the dermis of the wound returns to an architecture that is similar, although not identical, to normal skin.

The regulation of wound resolution remains incompletely understood, particularly the mechanisms that contribute to the ordered regression of capillaries. Prior studies in our lab have suggested that PEDF, a known endogenous anti-angiogenic protein, provides important signals that lead to capillary removal during this phase of repair^6^. The anti-angiogenic capability of PEDF has been best described in tissues other than skin, yet the mechanisms by which PEDF might influence capillary reduction in skin wounds are not yet well studied^7–10^.

The current study examines the effect of a global genetic deficiency of PEDF on wound healing *in vivo*, and the effect of PEDF on migration, tube formation, and the expression of key endothelial cell receptors by primary mouse dermal microvascular endothelial cells (MDMEC). The results demonstrate that the absence of PEDF leads to specific changes in the proliferative phase of healing, and causes a distinctive delay in wound resolution. The data further shows that PEDF has many direct effects on dermal microvascular endothelial cell function, and suggest a multi-factorial role for PEDF in the remodeling phases of dermal wound healing.

## Materials and Methods

### Animal Wound Models

All animal procedures were approved by the Institutional Animal Care and Use Committee (IACUC) of the University of Illinois at Chicago. The studies also adhered to the National Institutes of Health: *Guide for the Care and Use of Laboratory Animals*. Male and female 8–10 week old PEDF deficient mice (PEDF^−/−^) on a C57/BL/6 background^11^ and littermate wild type (WT) mice were housed in groups of three to five in a temperature-controlled environment (22 to 24 °C) on a 12-h: 12-h light-dark cycle and provided with food and water *ad libitum*. PEDF ^−/−^ mice and littermate WT mice were anesthetized via intraperitoneal injection of a ketamine (100 mg/kg) and xylazine (5 mg/kg) solution. The dorsal surface of each mouse was shaved using electric clippers and cleansed thoroughly with 70% isopropyl alcohol. For wound breaking strength studies, two (2 cm) incisional wounds were prepared on the dorsal skin as previously described^12^. All other studies utilized a standard well-described excisional wound model^13^. For excisional wound studies, a sterile 3-mm punch-biopsy instrument (Acu Punch, Acuderm, Ft. Lauderdale, FL) was utilized to make six excisional full-thickness dermal wounds on the dorsal surface of each mouse (three on each side of the midline). Skin that had been excised with the punch biopsy instrument served as normal, unwounded skin. Wound samples for mRNA analysis and protein analysis were obtained by excising wound samples with a 5 mm punch-biopsy instrument for wounds of days 1 and 3, and a 3 mm punch-biopsy instrument for wounds of days 5, 7, 10, and 21. Samples for RNA analysis were placed in RNAlater (Sigma-Aldrich, St. Louis, MO) and stored at −20 °C until use. Samples for protein analysis were snap frozen in liquid N_2_ and stored at −80 °C until use. Samples for histologic analysis were harvested with a 5 mm punch, placed in optimum cutting temperature (OCT) compound (Sakura Finetechnical, Tokyo, Japan), and snap frozen.

### Treatment of Wounds with rPEDF

Recombinant PEDF (rPEDF) was kindly provided by Dr. Peter Gettins, and was prepared as previously described^14^. Two 3 mm punch wounds were created on the dorsal skin of 8 week old female C57BL/6 mice (Jackson Laboratory, Bar Harbor, ME). Each wound was treated with topical rPEDF daily until day 10 post-injury. For the first 3 days, two µg rPEDF in 10 µl 25% pluronic gel (Sigma-Aldrich) was directly applied to the surface of each wound. From day 4 to day 10, 10ul 2 µg rPEDF in PBS was injected intradermally into each wound. Control wounds were treated with PBS. Wounds were harvested at days 7 and 10 post-injury, placed in RNAlater (Sigma-Aldrich) and kept at −20 °C until used. Some of the day 10 wound samples were embedded in OCT compound and later processed for CD31 immunofluorescent staining as described below.

### Immunofluorescent Histochemistry of Endothelial and Inflammatory Cells

For immunohistochemical studies, 8-μm thick cryo-sections were prepared from each wound sample. Samples were air-dried, re-hydrated in PBS, and fixed in cold acetone for 10 min. Specimens were then washed in PBS and blocked using a 10% normal goat serum (Sigma-Aldrich) followed by incubation at room temperature for 45 min with either rat anti-mouse Gr-1 (neutrophils) (BD Bioscience, San Jose, CA), rat anti-mouse CD68 (macrophages) (Abcam, Cambridge, MA), or rat anti-mouse CD31 (endothelial cells) (BD Pharmingen, San Diego, CA). Slides were then washed with PBS and incubated with the appropriate secondary antibodies including Alexa fluor 488 or 594 goat anti-rat IgG (Invitrogen, Carlsbad, CA) for 45 minutes. Stained sections were mounted with PBS containing DAPI for nuclear counterstaining and observed under a fluorescence microscope (Axioskop 40, ZEISS, Oberkochen, Germany) and recorded utilizing a digital camera (AxioCam HRc, ZEISS). Between 3 to 4 20x images were captured for each wound, including 1–2 images within the wound bed and one image at each wound margin. For quantification of inflammatory infiltrate, cells that were positively stained for CD68 and Gr-1 were counted in each field, and the average number of cells per 20x field was calculated. To quantify vascularity, the entire wound bed was traced, and the percent of the wound bed that was CD31+ was quantified using image analysis as previously described^15^. The analyses were performed by a blinded observer.

### Vascular Permeability Analysis

At day 7 post wounding, mice were anesthetized by intraperitoneal injection of 100 mg/kg of ketamine and 5 mg/kg of xylazine. 200 μL of FITC-conjugated high molecular dextran (15 mg/mL in PBS, Sigma-Aldrich) was injected into the retro-orbital venous plexus of each mouse 30 min before the mice were sacrificed. 5 mm biopsies were harvested from the wound site, snap frozen in OCT, sectioned, and stained for CD31. Images of sections at 20X magnification were analyzed using Fiji image processing software (http://fiji.sc), converted to 16-bit, and a threshold was applied with the Otsu algorithm in order to standardize the intensity level. The CD31 and FITC-dextran images were merged together to create a tri-color image indicating each respective color for CD31, dextran and their co-localization. The total area of the pixels was quantified and micro-vessel leakiness was measured as the percentage of extravascular dextran area in the wound bed over the total area as previously described^6^.

### Hematoxylin/eosin (H&E), Masson’s Trichrome, and Picrosirius Red Staining

Wounds were fixed in formalin, embedded in paraffin, sectioned and stained using H&E, Masson’s trichrome, and picrosirius red to evaluate histology, collagen content and maturity as described in our previous publication^6,16,17^. Slides were evaluated under a polarized microscope for picrosirius red staining. Photomicrographs were taken at 20X magnification. Total collagen deposition was quantified in Masson’s trichrome stained sections using FiJi by calculating the percent of blue-stained collagen area within the wound bed. Collagen maturity in picrosirius red stained sections was quantified using Fiji by calculating the amount of mature (red-orange) collagen as a percentage of total (red/orange + green) collagen. Total collagen was compared by calculating the percentage of the area occupied by either mature collagen or immature collagen.

### Wound Closure

External photographs of wounds were taken every other day during the time course of healing at a set distance from the camera and with the inclusion of a standard calibration scale. The size of each wound was analyzed in AxioVision. All six wounds were analyzed for each mouse and the average calculated to produce one unique value per time point per animal. To determine the percent wound closure, the wound area value from a given time point was divided by the original wound size using the formula: [(Average wound area)/(Original wound area)] × 100.

### Wound Breaking Strength

Wound breaking strength (WBS) of 2 cm incisional wounds from PEDF^−/−^ and WT mice was examined at day 14 post-wounding. Two standardized strips of skin that spanned the incisional wound were prepared from each mouse, and a motorized tensiometer (Mark-10, Copiague, NY) was used to determine WBS as previously described^12^. The breaking strength was then recorded as weight load (pound, lb) at the time that wound breakage occurred. The average was calculated for the two strips and was used as the WBS for that individual animal. The breaking strength of normal skin from six PEDF deficient and six PEDF wild type mice was analyzed as a control.

### Cell Culture and Migration Assay

MDMEC isolated from C57BL/6 mice (Cell Biologics, Chicago, IL) were used to test the effects of PEDF on migration, adhesion, proliferation, apoptosis, and tube formation. For the migration assay, cells were grown to confluence in 6-well plates and treated with mitomycin C (10 μg/ml, Sigma-Aldrich) for 2 hours to prevent cell proliferation. Scratch wounds were then created using 0.2 ml pipette tips. The cells were treated with a range of concentrations of human recombinant PEDF (1, 100, or 500 ng/ml) for 24 hours. Wounds areas were photographed at 0 and 24 hours and the wound size quantified in pixels using ImageJ^18^.

### Cell Adhesion Assay

To examine the effect of PEDF on cell adhesion, 5 × 10^3^ MDMEC were seeded in 96 well plate and incubated with varying concentrations of PEDF (1, 100, or 500 ng/ml) for 4 hours. Plates were washed to remove non-adherent cells. The remaining adherent cells were fixed in 10% ethanol and stained with 0.1% crystal violet for 5 minutes then lysed with 100 µl of 0.2%Triton x100 for 10 minutes. The OD_550_ values were determined using a spectrophotometer^19^. The percent adhesion was calculated as: OD_550_ remaining cells/OD_550_ untreated cells × 100.

### Assessment of Cellular Apoptosis

To determine if PEDF induces apoptosis in MDMEC, 6 × 10^4^MDMEC were seeded in 12 well plates for 24 hours and then treated with PEDF (100 ng/ml) or camptothecin (CPT, 5 µM/ml) as a positive control (Sigma-Aldrich). After 24 hours of treatment, the level of apoptosis was assessed using an Annexin V Apoptosis Detection Kit APC (eBioscience, Carlsbad, CA). Briefly, following the manufacturer’s directions, cells were stained for Annexin V and treated with propidium iodide (PI), and then subjected to flow cytometry analysis.

### *In vitro* Angiogenesis Assay

A standard tube formation assay was used to assess angiogenic capability. For each assay, 0.2 ml of chilled Matrigel Matrix (10 mg/ml, Corning Incorporated, Corning, NY) was placed in each well within a 48 well plate. Following a 30-minute incubation at 37 °C, 60 × 10^3^ MDMEC were added to each well with or without 100 ng/ml PEDF. Images of tube formation were obtained at 2 and 4 hours of incubation, times which were determined to be the time points of maximal tube formation for MDMEC. WimTube (Wimasis Image Analysis at wimasis.com) was used to analyze the tube formation. The parameters quantified using WimTube were: (1) Covered area (%), defined as the percentage of the image area covered by tubular structure, (2) Total tube length, defined as the length in pixels of the all tubular structures in the image, (3) Total tubes, defined as the number of independent tubular structures located between two branching points or a branching point and a loose end, (4) Total branching points, defined as location within the image where three or more tubes converge, (5) Total loops, defined as the percent of the image area enclosed by tubular structures.

### Flow cytometry

MDMEC were grown to 70–80% confluence in 6-well plates and treated with human rPEDF (100 ng/ml) for 24 hours. Cells were then dissociated from the plate using TrypLE Select (Gibco/Thermo Fisher Scientific, Waltham, MA). Single cell suspensions were incubated with anti-mouse CD31 (PECAM-1)-PE-Cy7 (Invitrogen), anti-CD106 (VCAM-1)-eFluor 450 (Invitrgoen), anti-CD62E (E-Selectin)-PE (Miltenyi, Bergisch Gladbach, Germany), anti-CD62P (P-Selectin)-APC (Miltenyi), and anti-CD309 (VEGFR2)-APC-Vio770 (Miltenyi) for 15 min on ice. All antibodies were used at the concentration of 0.5 μg/ml. After washing, stained cells were analyzed using a Cyan cytometer (Dako Cytomation, Glostrup, Denmark). Results were analyzed by Summit 4.3 software (Dako Cytomation).

### Real time PCR

To assess mRNA expression of CD31, VEGFR2, VCAM-1, E-Selectin and P-Selectin in PEDF treated MDMEC, MDMEC were grown to 70–80% confluence and treated with 0 or 100 ng/ml of PEDF for 4 hours. Total RNA was extracted from cells using TriZol (Invitrogen, Carlsbad, CA). For mRNA expression of IL-1β, IL-6, TNF-α, CD31, VEGFR2, VCAM-1, E-Selectin and P-Selectin within *in vivo* wounds, tissues were homogenized and total RNA was extracted from the wound tissue using TriZol (Invitrogen) as previously described^16^. Both cell derived and wound tissue RNA samples were treated with DNAse I and subjected to reverse transcription utilizing a RETROscript kit (Invitrogen) as previously described^20^. Quantitative PCR was performed using a real-time PCR system (StepOne Plus; Applied Biosystems, Carlsbad, CA), which employs SYBR Green PCR mix (Roche, Basel, Switzerland) as well as gene specific primers. Glyceraldehyde 3-phosphate dehydrogenase (GAPDH) was used for normalization. Primer sequences are listed in Table 1.

**Table 1:** Primers sequences for real time PCR.

Genes	Forward (5′-3′)	Reverse (5′-3′)
CD31	GAGCCCAATCACGTTTCAGTT T	TCCTTCCTGCTTCTTGCTAGCT
E-Selectin	TTTCAATGCAATGAGGGCTTT	GGACGTCAAGGCTTGGACAT
Fas	CCTCCAGTCGTGAAACCATAC	TCTTGCCCTCCTTGATGTTATT
GAPDH	TCACCACCATGGAGAAGGC	GCTAAGCAGTTGGTGGTGCA
IL-1β	CAACCAACAAGTGATATTCTCCATG	GATCCACACTCTCCAGCTGCA
IL-6	GAGGATACCACTCCCAACAGACC	AAGTGCATCATCGTTGTTCATACA
P-Selectin	GCCAGTTCATGTGCGATG	GGCGAAGATTCCTGGACACTT
TNF-α	CATCTTCTCAAAATTCGAGTGACAA	TGGGAGTAGACAAGGTACAACCC
VCAM-1	GTGAAGATGGTCGCGGTCTT	GGCCATGGAGTCACCGATT
VEGFR2	GCGGAGACGCTCTTCATAATA	GACAAGAAGGAGCCAGAAGAA

### Statistical analysis

Results are expressed as means ± standard deviations (SD). The “n” for all animal experiments indicates the number of unique mice whose wounds were analyzed. If more than one wound sample was analyzed from a single mouse, the replicate values were averaged to create a unique value for each mouse. Two-way ANOVA followed by Bonferroni’s post-hoc analysis or two- tailed Student’s *t-*tests were performed using GraphPad Prism 6.0 software (GraphPad Software, San Diego, CA). P values less than 0.05 were considered statistically significant.

## Results

### The absence of PEDF causes a transient early impairment in wound closure

**Figure 1:**
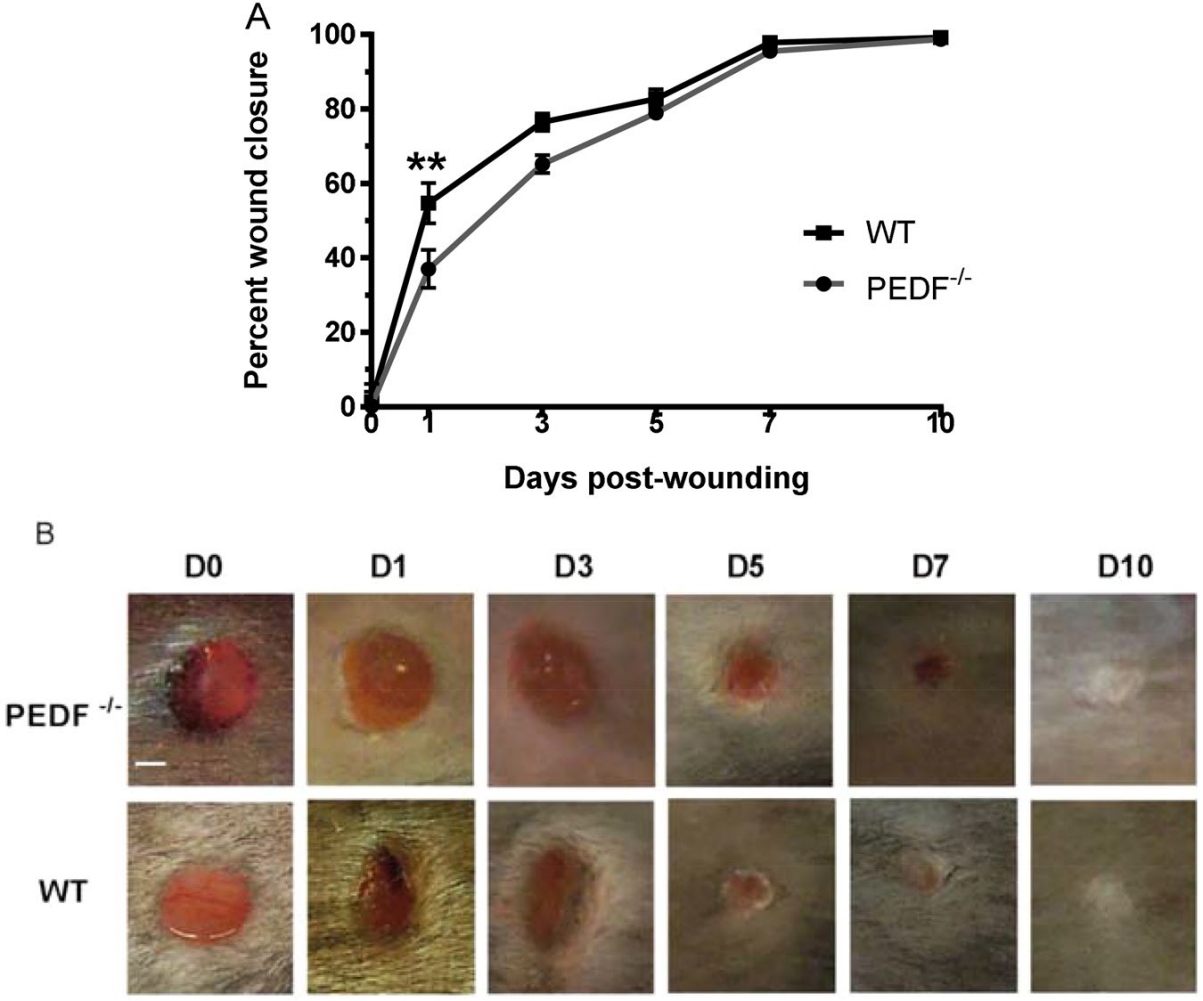
Wound closure rates in PEDF^−/−^ and WT mice. (**A**) Wound closure, normalized to original size of wound bed and expressed as mean ± SD; n = 4 for PEDF^−/−^ Day 10 and WT Days 7 & 10; n = 6 for PEDF^−/−^ Day 7 and WT Days 1, 3, & 5; n = 7 for PEDF^−/−^ Days 0, 1, 3, & 5; n = 9 for WT Day 0; *p < 0.05 by two-way ANOVA followed by Bonferroni’s post-hoc analysis. (**B**) Representative photomicrographs showing the time course of wound healing. Because hair was removed using clippers, hair removal occasionally appears uneven. Scale bar = 1 mm.

To examine how the loss of PEDF influences epithelial closure, external wound closure was quantified and compared between PEDF^−/−^ and WT mice at days 1, 3, 5, 7 and 10. Wound closure was slightly delayed in PEDF^−/−^ mice compared to WT, with a significant delay only at day 1 (p < 0.01) (Fig. 1). All wounds in both WT and PEDF^−/−^ mice closed on day 7 post-wounding as demonstrated by H&E stained wound sections (Fig. 1C). Thus, the loss of PEDF causes a transient but recoverable disruption to epithelial closure.

### Wounds of PEDF^−/−^ mice show changes in vascular content and permeability

**Figure 2:**
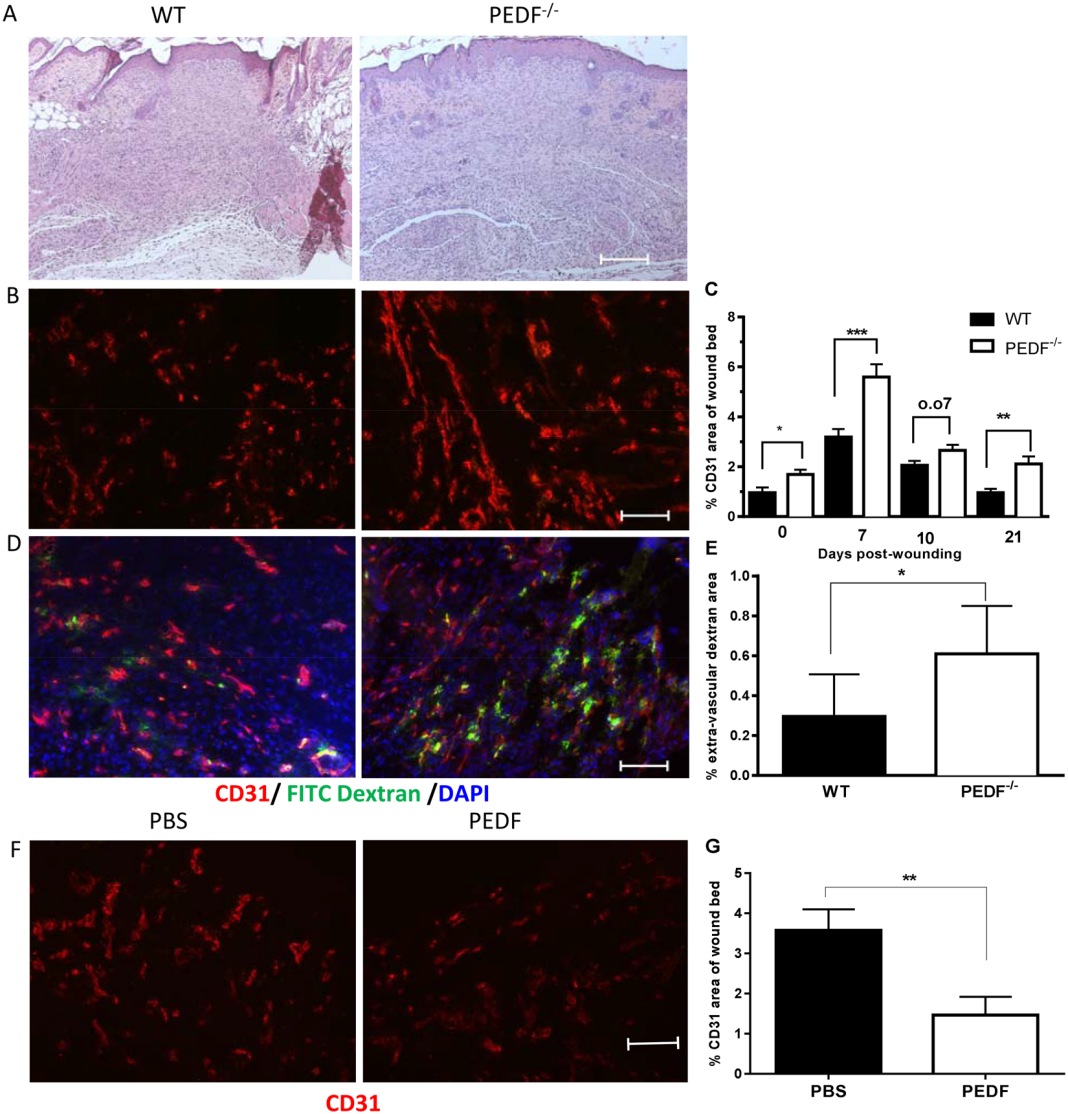
Capillary content and permeability in WT, PEDF^−/−^ or PEDF treated mice. (**A**) Representative photomicrographs of the histology of day 7 wound (H&E staining). Skin wounds in both groups closed at day 7. (**B**) Representative fluorescence photomicrographs of CD31 staining in wounds. Scale bar = 100μm. (**C**) Wound vascularity, quantified by measuring the percent area of wound bed occupied by CD31 staining. Mean ± SD, n = 5 for normal unwounded skin (NS)- WT, Day 10- WT, Day 21- WT and Day 21- PEDF^−/−^; n = 6 for Day 7- PEDF^−/−^ and Day 10- WT; n = 7 for NS- PEDF^−/−^ and Day 10- PEDF^−/−^; *p < 0.05 versus NS, **p < 0.01, ***p < 0.001 by two-way ANOVA and Bonferroni’s post-tests. (**D**) Vascular permeability in day 7 wounds of PEDF^−/−^ and WT mice following perfusion with FITC-dextran 30 min before wound harvest. Representative fluorescence photomicrographs of CD31 (red) and dextran (green) staining. (**E**) Blood vessel permeability quantified as the percent area of extra-vascular dextran (dextran not co-localized with CD31) in the wound bed at day 7 after injury. Mean ± SD; n = 5 for WT mice and n = 6 for PEDF^−/−^. *p < 0.05 by Student t-test. (**F**,**G**) Effect of exogenous PEDF treatment on wound vascularity. (**F**) Representative fluorescence photomicrographs of CD31 staining in day 10 wounds after PEDF treatment. Scale bar = 100μm. (**G**) Quantification of wound vascularity at day 10, measured as percent area of wound bed occupied by CD31 staining. Mean ± SEM, n = 5 for both groups, **p < 0.01 by Student t-test.

Given the expected influence of PEDF on wound angiogenesis, we next examined vascular content and permeability in skin and wounds of PEDF^−/−^ and WT mice. Consistent with its presumed role as an anti-angiogenic mediator in skin, immunohistochemical quantification of CD31+ capillaries revealed a statistically significant increase in the number of vessels in PEDF^−/−^ vs WT mice (Fig. 2A,B). This increased vascularity was observed both in normal skin (NS) (p < 0.05) and at day 7 post injury (p < 0.001) which is a period of active angiogenesis^6^. Vascularity in the wounds of PEDF^−/−^ mice was increased at day 10 (p = 0.07) and at day 21 post injury (p < 0.01), time points at which capillary pruning occurs in skin wounds (Fig. 2C). During the proangiogenic phase, days 0–7, the wounds of the PEDF^−/−^ mice exhibited 1.75 times more net growth of capillaries than that seen in WT (3.91 versus 2.23 increase in % CD31+ area). During the anti-angiogenic phase, days 10–21, the wounds of the PEDF^−/−^ mice exhibited a reduced amount of vascular regression as compared to the WT mice (0.55 versus 1.1 decrease in % CD31+ area). Overall, the net change in capillary content in PEDF^−/−^ versus WT mice is consistent with a role for PEDF as an inhibitor of angiogenesis and a mediator of vessel regression. To determine if PEDF influences capillary leakage in wounds, vessel permeability was examined by IV injection of FITC- conjugated high molecular weight dextran and quantification of FITC dextran leakage into wounds. Wounds of PEDF^−/−^ mice exhibited a statistically significant increase in vessel permeability as compared to WT mice (p < 0.05) at day 7 (Fig. 2D). Overall, wounds of PEDF^−/−^ mice exhibited more exuberant angiogenesis, a delay in vascular pruning, and increased vessel permeability. To further demonstrate that PEDF can negatively affect wound angiogenesis, wounds of WT mice were treated with recombinant PEDF. The results of these studies show that rPEDF treatment significantly inhibits wound angiogenesis, providing further confirmation that PEDF can function as a negative regulator of wound angiogenesis (Fig. 2F,G).

### PEDF deficiency does not alter the inflammatory response in wounds

**Figure 3:**
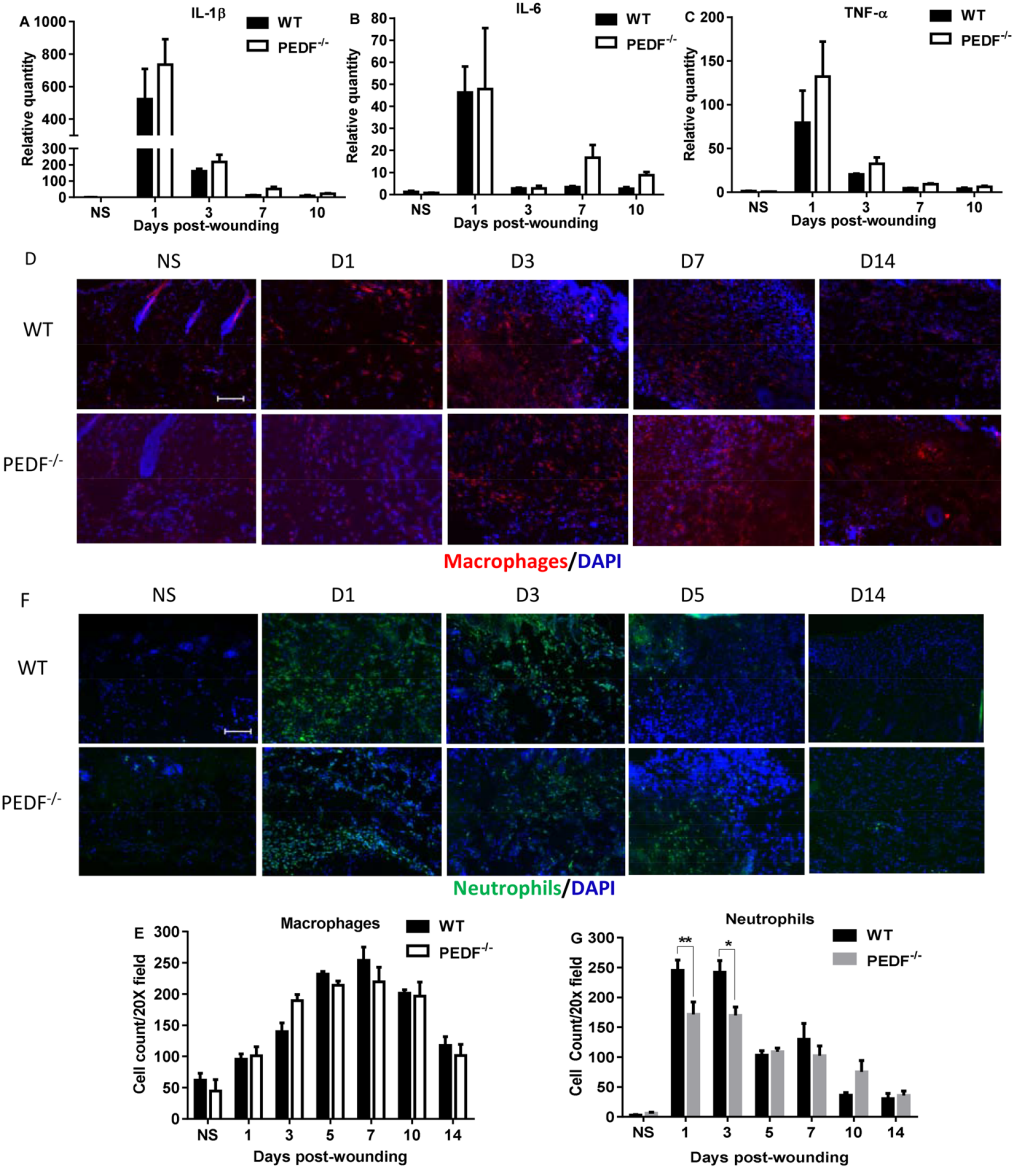
Analysis of inflammatory cytokine expression and immune cell content in wounds of PEDF^−/−^ and WT mice. (**A**–**C**) Levels of mRNA expression of the inflammatory cytokines IL-1β, IL-6, and TNF-α. Mean ± SEM; n = 5 in all groups and at all time points except n = 4 for WT Day 3 and PEDF^−/−^ Day 7. (**D**) Representative images of macrophage (CD68) staining at selected time points in wounds of PEDF^−/−^ and WT mice. (**E**) Macrophage cell counts in wounds of PEDF^−/−^ and WT mice. Mean ± SD; n = 3 for WT Days 5 &14 and PEDF^−/−^ Day 5; n = 4 for WT Day 3 and PEDF^−/−^ Day 7; n = 5 for WT NS, Days 7&10, and PEDF^−/−^ Days 1, 3 &14; n = 6 for WT Day1 and PEDF^−/−^ NS and Day 10. (**F**) Representative images of neutrophil (Gr-1) staining at selected time points in wounds of PEDF^−/−^ and WT mice. (**G**) Neutrophil cell counts in wounds of PEDF^−/^ and WT mice. Mean ± SD; n = 3 for WT Days 5 &14; n = 4 for WT Day 3 and PEDF^−/−^ Days 5 &10; n = 5 for WT Days 7&10 and PEDF^−/−^ Days 7 &14; n = 6 for WT NS; n = 7 for WT Day 1; n = 8 for PEDF^−/−^ NS & Day 1; n = 10 for PEDF^−/−^ Day 3. *p < 0.05 and **p < 0.01 by two-way ANOVA followed by Bonferroni’s post-hoc analysis. Scale bar = 100 µm.

Since inflammation is known to influence wound healing, and because increased inflammation is often linked to the angiogenic response, we next examined whether changes in inflammation might be involved in the observed changes in angiogenesis seen in PEDF^−/−^ mice. PEDF^−/−^ mice displayed slightly higher amounts of IL-1β, IL-6, and TNF-α mRNA at several time points, but these differences did not reach statistical significance (Fig. 3A–C). The number of inflammatory cells, including neutrophils and macrophages, was also examined in wounds (Fig. 3D,E). Compared to WT, wounds from PEDF^−/−^ mice exhibited significantly lower levels of neutrophils at days 1 and 3. No significant differences were seen in macrophage content. These results suggest that the increased angiogenesis seen in PEDF^−/−^ mice does not derive from altered inflammation, but is most likely a direct result of the loss of PEDF anti-angiogenic activity on endothelial cells themselves.

### Changes in collagen content and maturity occur in wounds of PEDF deficient mice

**Figure 4:**
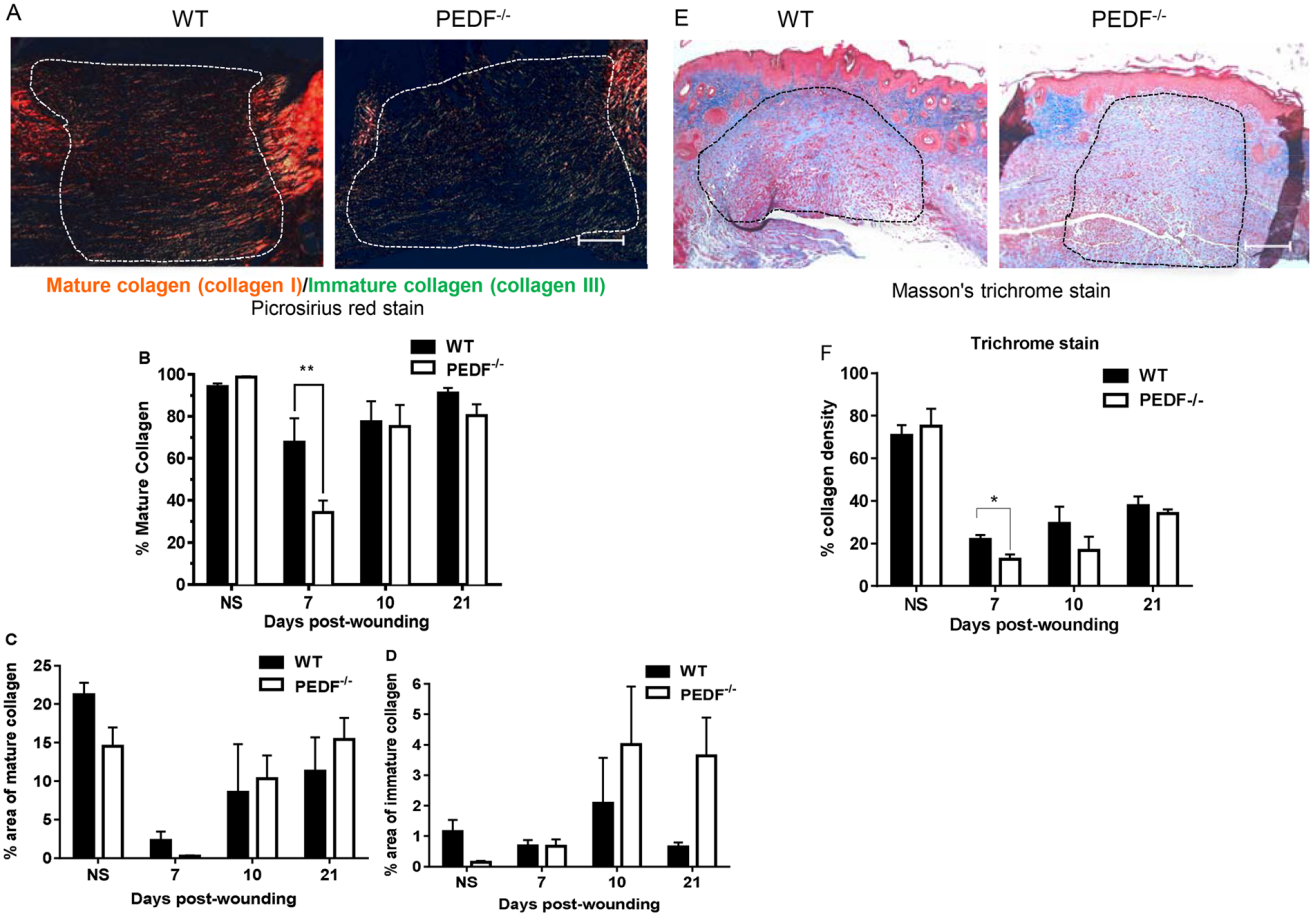
Assessment of collagen content and maturity in wounds of WT vs. PEDF^−/−^ mice. (**A**) Representative photomicrographs of picrosirius red stain demonstrate the distribution of green-yellow (immature) and red-orange (mature) collagen in day 7 wounds. Scale bar = 100μm. (**B**) Percentage of mature collagen in the wound bed, calculated as (area of red-orange stain)/(total area of collagen) based on picrosirius red staining. Mean ± SD, n = 5 for all time points, except n = 4 at Day 10- PEDF^−/−^ and n = 6 for NS-WT and NS- PEDF^−/−^. **p < 0.01 by two-way ANOVA followed by Bonferroni’s post-hoc analysis. (**C**) Percentage of area occupied by mature collagen, not significant. (**D**) Percentage of area occupied by immature collagen based on picrosirius red staining, not significant. (**E**) Representative photomicrographs of Masson’s trichrome stain demonstrate the distribution of collagen (blue) in day 7 wounds. Scale bar = 100μm. (**F**) Percentage of area occupied by collagen based on Masson’s trichrome staining. Wound bed areas marked by dotted lines were used for quantification. Mean ± SD, n = 3 for WT NS & Day 10 and PEDF^−/−^ Day 21; n = 4 for PEDF^−/−^ Day 7; n = 5 for WT Day 21 and PEDF^−/−^ NS; n = 6 for WT Day 7 & PEDF^−/−^ Day 10. *p < 0.05 by two-way ANOVA followed by Bonferroni’s post-hoc analysis.

To examine whether the absence of PEDF affects extracellular matrix synthesis and remodeling, collagen maturity was assessed in skin wounds of PEDF^−/−^ and WT mice. Time points that correlate to active angiogenesis (day 7 and 10), granulation tissue formation (day 10) and collagen remodeling (day 21) were examined. As assessed by quantification of picrosirius red staining, a statistically significant decrease in percent of Type 1 collagen was seen in PEDF^−/−^ vs. WT mice at day 7 post wounding (p < 0.01) (Fig. 4A,B). The lag in collagen maturity ended by day 10, as wounds of PEDF^−/−^ and WT mice showed similar collagen maturity by day 10 (Fig. 4A,B). The area occupied by mature, type 1 collagen or immature, type 3 collagen was not statistically significantly different between wounds of PEDF^−/−^ and WT mice at any time point (Fig. 4C,D). However, as compared to WT, wounds of PEDF^−/−^ demonstrated a trend toward increased immature type 3 collagen at days 10 and 21 (Fig. 4C,D). Total collagen deposition, assessed using Masson’s trichrome stain, was significantly reduced in day 7 wounds of PEDF^−/−^ mice (Fig. 4E,F), which confirmed the results described in picrosirius red staining. As another assessment of ECM maturation, WBS was compared between PEDF^−/−^ and WT mice. WBS is a well-accepted and clinically relevant marker of ECM reconstitution in wounds. To determine if the absence of PEDF causes measurable differences in dermal reconstitution, WBS was measured in PEDF^−/−^ and WT mice at day 14 after wounding. As expected, WBS was significantly less than the breaking strength of uninjured normal skin for both strains of mice (p < 0.0001). However, no difference in breaking strength was seen between WT and PEDF^−/−^ strains in either unwounded skin (1.43 ± 0.26 lb vs. 1.33 ± 0.27 lb, NS) or day 14 wounds (0.48 ± 0.10 lb vs. 0.50 ± 0.11 lb, NS). Together, the observations suggest that the loss of PEDF creates a time-limited delay in collagen maturity during the early stages of repair. By day 10–14, wounds from PEDF deficient mice have overcome this delay in collagen development, and no longer exhibit any deficit in collagen maturity or WBS.

### PEDF induces apoptosis and inhibits migration of dermal endothelial cells

**Figure 5:**
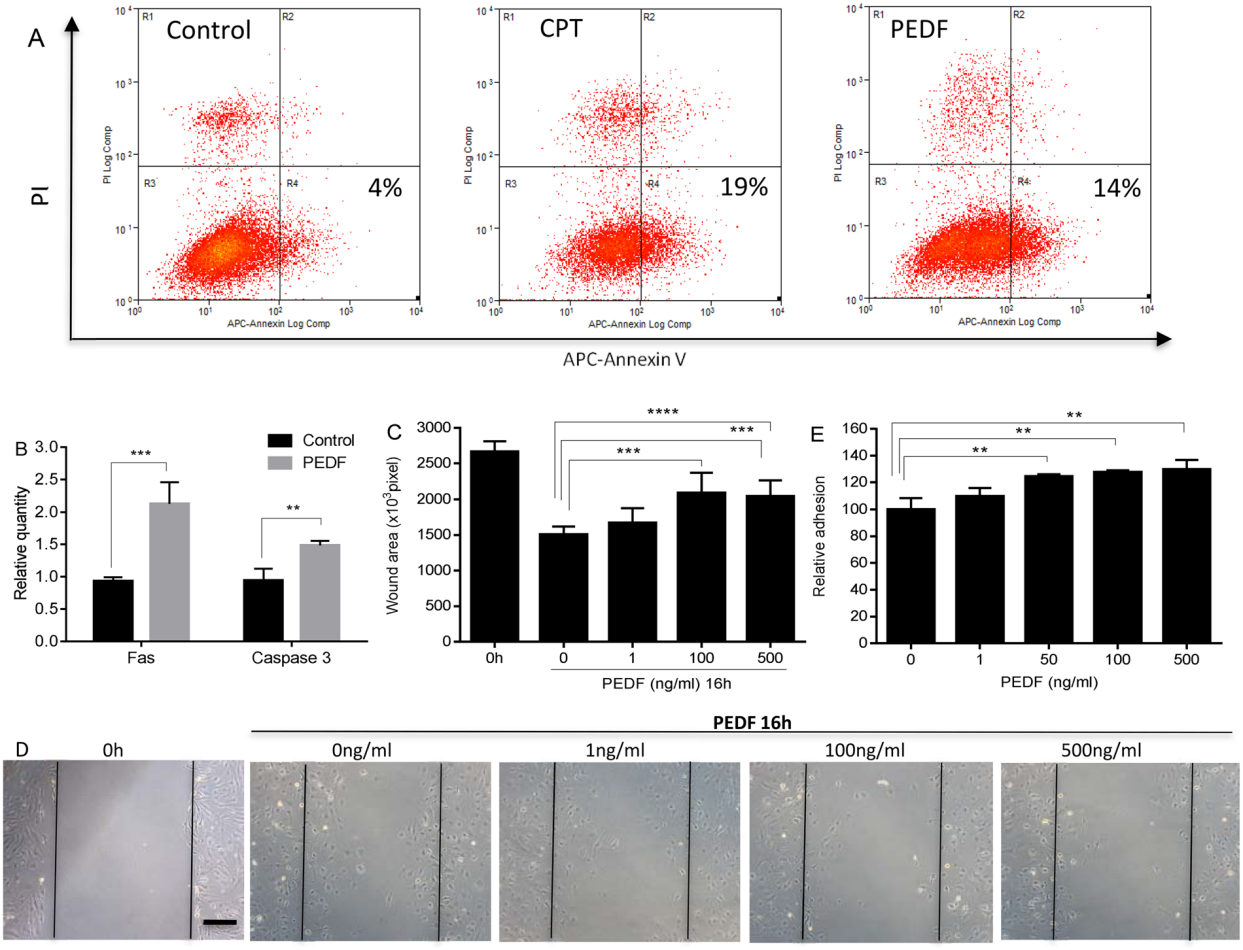
Dose response effect of PEDF on apoptosis, migration, and adhesion of mouse dermal endothelial cells (MDMEC). (**A**) Representative flow cytometric analysis of MDMEC exposed to PEDF or CPT. MDMEC were treated with PEDF (100 ng/ml) or CPT (5uM/positive control) for 24 hours. Early apoptotic cells were detected by flow cytometry using an annexin V apoptosis detection Kit. (**B**) Fas and caspase 3 mRNA expression after PEDF treatment. n = 3 for control Fas & caspase 3, and PEDF caspase 3; n = 6 for PEDF Fas. (**C**) *In vitro* migration of MDMEC following scratch wound placement with/without PEDF treatment. Wound area was quantified in pixels using ImageJ. Mean ± SD; n = 5 for 0 h, n = 5, 12, 12, and 15 for PEDF 0, 1, 100, and 500 ng/ml, respectively. (**D**) Representative images of cell migration in scratch wounds. Scale bar = 100 µm. (**E**) Adhesion of MDMEC with and without PEDF, assessed by measuring the quantity of remaining adherent cells following a 4 h exposure to PEDF. Mean ± SD; n = 3. **p < 0.01, ***p < 0.001, and ****p < 0.0001, Student’s t-tests was used for statistical analysis.

As the most dramatic changes in skin wounds in the PEDF^−/−^ mice were those related to capillary content and permeability, the ability of PEDF to directly influence dermal endothelial cell function was examined *in vitro*. Prior studies of EC from tissues other than skin have shown that PEDF causes EC to undergo apoptosis^21–23^. To determine if this effect might be similar in skin, MDMEC were exposed to PEDF and apoptosis assessed. The exposure of MDMEC to PEDF led to rates of apoptosis similar to that seen for camptothecin, a known promoter of apoptosis (Fig. 5A). PEDF was found to significantly increase the number of cells in the early apoptotic stage (Annexin V+/PI+) (14% PEDF, 19% CPT, 4% control untreated) (Fig. 5A). An examination of the mRNA expression of two downstream markers of apoptosis, Fas and caspase 3, further demonstrated that PEDF treatment of MDMEC significantly upregulated the expression of both markers (Fig. 5B). Using *in vitro* assays, the effect of PEDF on the functional attributes of migration and adhesion of primary EC was investigated. In a standardized scratch assay, PEDF treatment significantly inhibited MDMEC migration in a dose-dependent manner (Fig. 5C). PEDF led to a dose dependent increase of endothelial cell adhesion, an effect that might contribute to decreased migration (Fig. 5D). These results are consistent with the effects of PEDF on other endothelial cells from sources such as the retina and prostate^7,11^.

### PEDF inhibits endothelial cell tube formation *in vitro*

**Figure 6:**
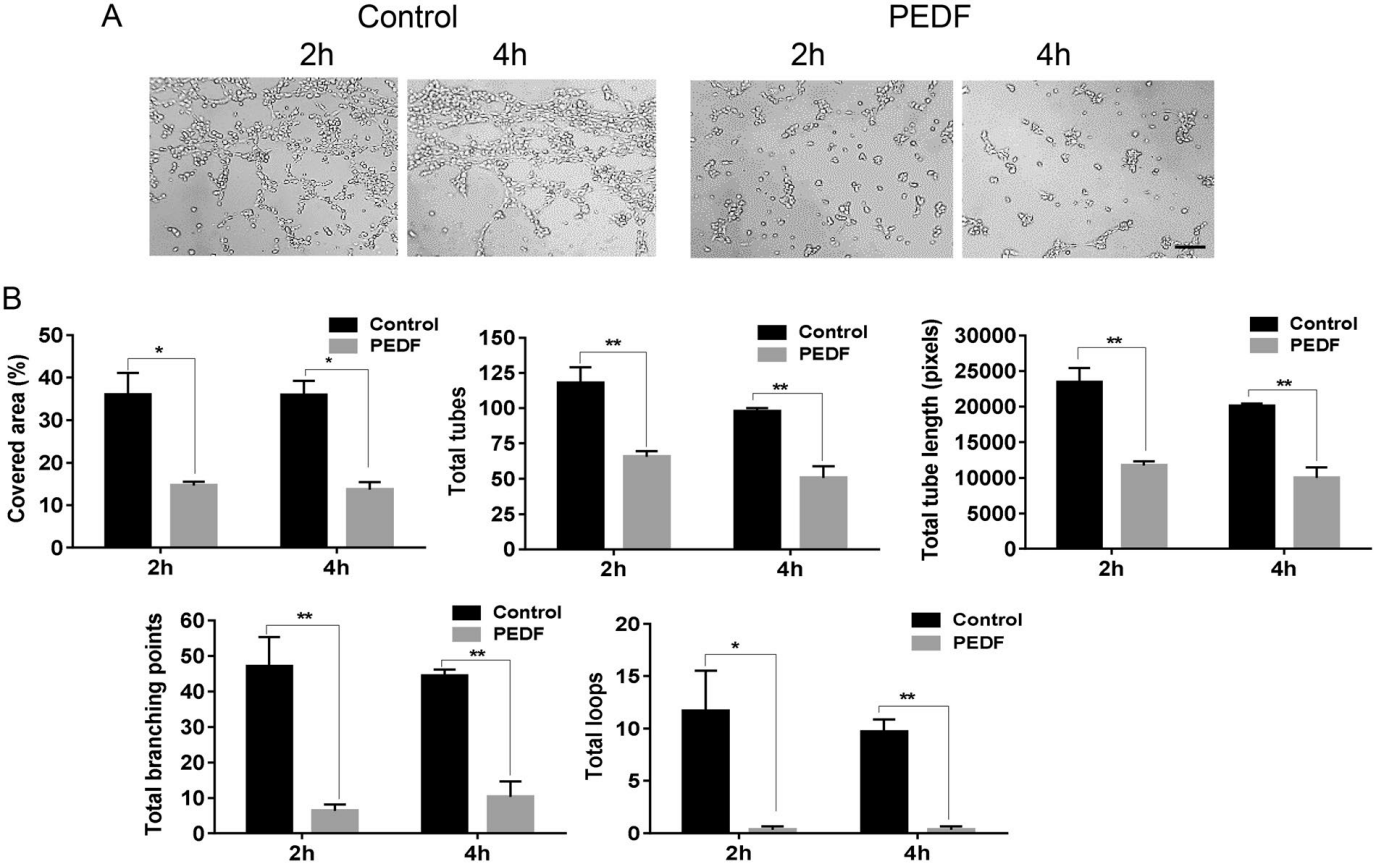
Effect of PEDF on endothelial cell tube formation. MDMEC were cultured on Matrigel with or without PEDF (100 ng/ml). (**A**) Images of tube formation at 2 and 4 hours after incubation. (**B**) WimTube software was used to quantify the parameters of tube formation including covered area, total tube length, total tubes, total branching points, and total loops. *p < 0.05, **p < 0.01, n = 3, Student’s t-tests was used for statistical analysis. Scale bar = 50 µm.

The ability of EC to form tubes on Matrigel is a commonly used assay for angiogenic capability. MDMEC were placed on Matrigel in the presence or absence of PEDF, and tube formation was assessed at 2 and 4 hours. Treatment of MDMEC with PEDF led to significant reductions in total tube length, total tubes, total branching points, and total loops as compared to the untreated control at both 2 and 4 hours (p < 0.05–0.01) (Fig. 6A,B).

### PEDF alters the expression of multiple endothelial cell surface molecules

**Figure 7:**
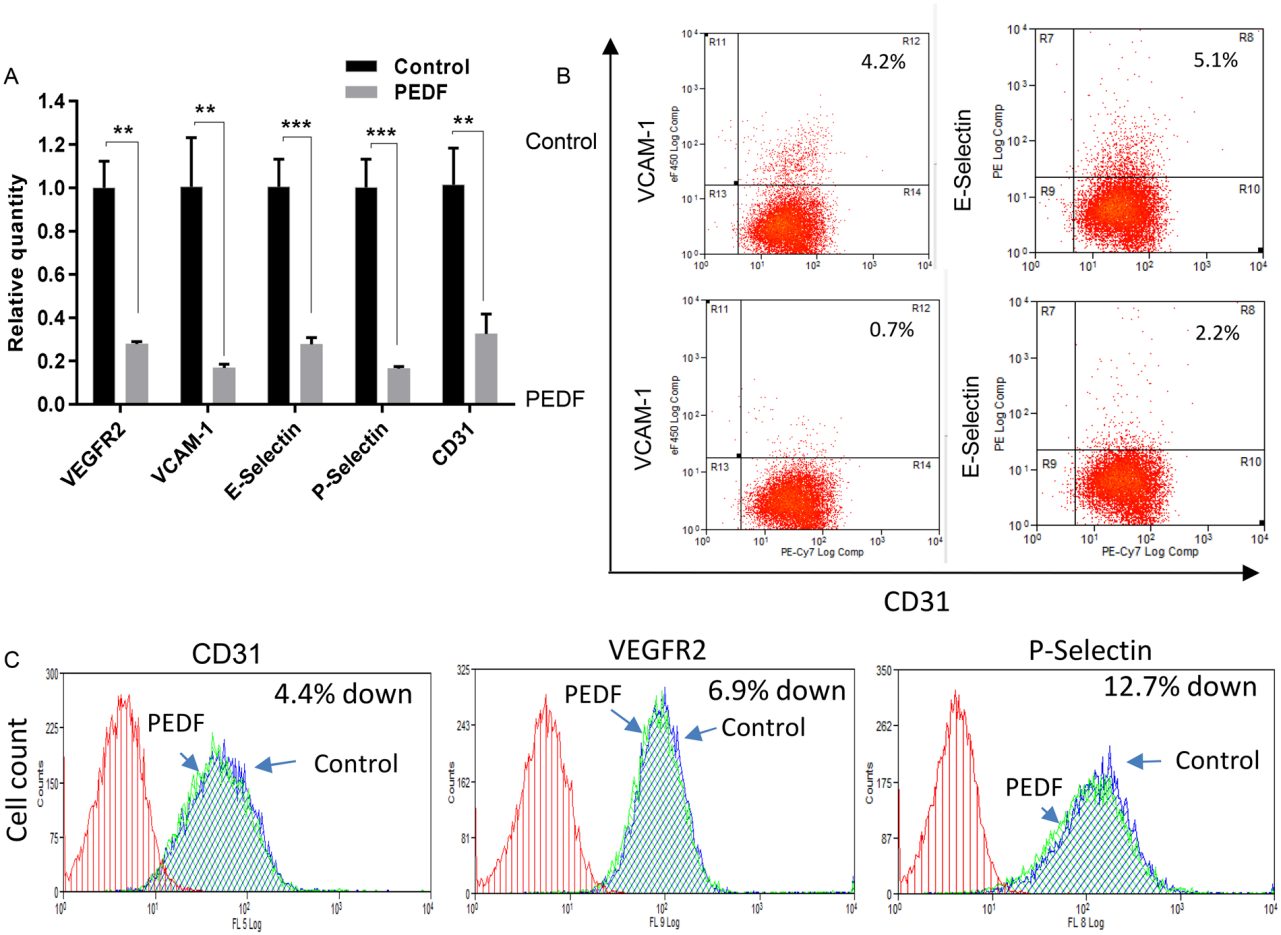
PEDF regulation of the expression of endothelial cell markers and surface receptors. (**A**) The effect of PEDF (100 ng/ml) treatment of MDMEC on the relative mRNA expression of CD31, VEGFR2, VCAM-1, E-Selectin, and P-Selectin as determined by semi-quantitative real time PCR 4 hours after PEDF treatment. The average value for untreated cells (control) was set at 1. Mean ± SD; n = 3. **p < 0.001, ***p < 0.001, Student’s t-tests was used for statistical analysis. (**B**,**C**) Representative flow cytometry analysis of endothelial cell markers and surface receptors 24 hours after PEDF treatment. Similar results were obtained in another experiment.

To further understand how PEDF might reduce angiogenesis and cause capillary regression in wounds, the effect of PEDF on the mRNA expression of a panel of cell surface molecules known to be important to endothelial cell function was examined. Treatment of MDMEC with PEDF (100 ng/ml for 4 hours) greatly suppressed the expression of all surface molecules that were examined, including the angiogenic receptor VEGFR2 and VCAM-1, a surface molecule that is critical to capillary formation and stability (Fig. 7A). Cellular expression of VCAM-1 and E-Selectin on endothelial cells were 83%, and 57% downregulated 24 hours after PEDF treatment compared to control (Fig. 7B). Since nearly all cells were CD31, VEGFR2 and P-Selectin positive, we use mean fluorescence intensity (MFI) to quantify the protein expression levels of those markers. As shown in Fig. 7C, MFIs for CD31, VEGFR2 and P-Selectin 24 hours after PEDF treatment were downregulated 4.4%, 6.9%, and 12.7%, respectively, compared to control. *In vivo*, the expression CD31, VEGFR2, VCAM-1, and E-Selectin were similarly decreased following the administration of exogenous PEDF (Supplementary Fig. 1). Although the *in vivo* response was more moderate than what was observed in PEDF treated MDMEC *in vitro*, the combined results suggest that at least part of the mechanism by which PEDF influences wound repair involves the direct down-regulation of surface receptors on endothelial cells.

## Discussion

The findings presented here provide new information about the role of PEDF in wound healing. Although the function of PEDF has been studied in several different processes, an appreciation of a role for PEDF in wound healing is relatively recent. The *in vivo* experiments here utilized a genetically deficient model to investigate PEDF’s role during the inflammatory, proliferative, and remodeling phases of wound healing. The factors that regulate wound resolution, including the pruning of capillaries and the remodeling of the ECM, are not well described. Although a few candidate factors, including CXC3 receptor, ECRG4, thrombospondins, and Sprouty-2, have been proposed to inform healing resolution outcomes^24–27^, many other factors are suspected to play a role in regulating the resolution phase of healing. The current study shows that the loss of PEDF significantly impairs the ability of the wound to resolve, establishing a genetic wound healing phenotype that is unique. PEDF, a member of the serine proteinase inhibitor (Serpin) family^7^, is a 50 kDa extracellular protein that is characterized by its neurotrophic, anti-tumorigenic, and importantly, its anti-angiogenic properties^9^. PEDF is produced by quiescent fibroblasts in a variety of tissues, including human skin, and is found at physiological levels in human blood^28–30^.

Previous studies demonstrate that PEDF levels increase during the resolving phase of dermal wound healing and vessel regression^6^. While the trigger for the rise in this anti-angiogenic stimulus is not yet known, recent studies suggest that TGF-β could provide the signal, as TGF-β stimulates PEDF production via pathway that involves decreased fibroblast Cav-1 expression^31^. Other possible triggers for PEDF production in wounds include changes in hypoxia, which inhibits PEDF production and decreases as capillary ingrowth increases^32^. Exposure to the plasminogen kringle 5 domains in wounds may also spur PEDF production^33^.

The current study suggests that the most significant effect of PEDF in skin wounds is its ability to specifically target neovessels and reduce vascular content in wounds without disrupting intact mature vasculature. The complete absence of PEDF led to an increase in wound capillary content in PEDF^−/−^ vs WT mice during the proangiogenic phase of healing and the capillary regression phase. Thus, PEDF function in wounds appears to be bimodal in importance. During the proangiogenic phase of repair, the vessels that are formed are frequently poorly organized and tortuous in architecture, creating a tangled and poorly perfused provisional capillary bed. PEDF may limit the initial angiogenic response in wounds, thus reducing the number of poorly perfused vessels in the neo-capillary bed. This concept is supported by our finding that mice that were deficient in PEDF also exhibited a significantly increased level of vascular permeability. In wounds, PEDF may limit the growth of initial immature and more permeable capillaries, thus reducing edema, and consequently reduce the capillary pruning requirement in the resolution phase. Our findings show that the wounds of PEDF^−/−^ mice maintained higher levels of capillaries for longer periods, supporting prior suggestions that PEDF is an important mediator of wound resolution^6^.

The mechanisms that underlie the effect of PEDF on wound angiogenesis appear to be multifactorial. PEDF’s ability to induce endothelial cell apoptosis has been demonstrated in prior studies of EC from tissues other than skin^34–36^. The current study extends this function to skin wounds, and provides evidence of the importance of PEDF in the healing skin wound. Our *in vitro* studies suggest that PEDF has many direct effects on dermal endothelia cells, including inhibition of migration, adhesion, and tube formation. Our studies suggest that many of these effects might derive from specific down regulation of important surface receptors. PEDF treatment led to a significant downregulation of the expression of both angiogenic receptors and other surface receptors dermal EC. In aggregate, then, PEDF exerts multiple direct biological effects on endothelial cells that might be important to its regulatory role in wound angiogenesis.

In addition to direct effects on the angiogenic response and capillary permeability, our findings support the possibility of additional less striking roles for PEDF in healing wounds, including a time-based modification of collagen synthesis. This finding is in keeping with emerging evidence that PEDF can influence multiple cell types other than EC. Of possible importance to skin wounds, PEDF has been shown to be produced by and chemotactic for fibroblasts^37,38^. Here we show that the loss of PEDF leads to a delay in the development of a mature collagen matrix in wounds, a fact that may derive from a direct of effect of PEDF on fibroblasts^38^, such as those reported in other systems. For example, PEDF has been shown to be a chemoattractant for wound fibroblasts, a function that would couple the resolution of angiogenesis with the restoration of a durable dermis^38^. In liver fibrosis models, PEDF can modify fibrosis via the regulation of the PDGF receptor in hepatic stellate cells^39^. Whether PEDF influences wound fibroblasts directly is not yet clear. Even if so, the impact of PEDF deficiency on collagen synthesis and remodeling in wounds seems quite transitory, as the wounds of the PEDF deficient mice ultimately healed with normal collagen content. Nevertheless, such effects might be quite important to ideal repair responses, or could be of larger importance in poorly healing or scar forming wounds. Interestingly, recent studies in our lab have demonstrated that the addition of exogenous recombinant PEDF can decrease scar formation and lead to increased levels of Collagen Type I^6^. Together with the current study, these findings suggest that PEDF may play an important role in wounds not only by influencing capillaries, but also by modulating fibroblast function, the production of ECM, and ultimately, scarring outcomes.

PEDF has also been shown to have direct effects on keratinocytes, and a modest transient negative effect on epithelial repair was observed in the PEDF^−/−^ mice. Prior studies of the effect of PEDF on keratinocytes are conflicting, but at least one recent study demonstrates that PEDF can stimulate keratinocyte proliferation^19,20,40^. At the early stage of healing this positive of effect of PEDF would be lost in the PEDF^−/−^ mice, resulting in delayed epithelial closure. At later time points, the dominate factors that mediate keratinocyte proliferation and epithelial closure, such as KGFs, are likely to overcome any negative effect of the loss of PEDF on epithelial repair^41^. Such a situation could explain why the loss of PEDF would have only a minor impact on the epithelialization process. Such a transient delay is unlikely to be biologically significant, reinforcing the concept that the most important role of PEDF in healing wounds of normal animals is not related to any direct effect on epithelium. In contrast to the current findings, a previous study that employed a model of diabetic healing showed that in the context of diabetes, loss of PEDF led to accelerated wound closure^42^. In this case, though, the effect of PEDF on epithelial closure resulted from the high circulating levels of PEDF seen in diabetes rather than PEDF produced at the wound site. High levels of circulating PEDF in diabetes could inhibit early epithelialization, and have also been suggested to cause a decrease in circulating endothelial progenitors, shutting down the angiogenic process and contributing to impaired healing seen in diabetics^42,43^. Thus the levels, role, and effect of PEDF on wound healing and wound closure may be very different in normal and diabetic mice.

One important finding of current study is that the influence of PEDF as an anti-angiogenic mediator in wounds was found to be independent of inflammation. The inflammatory phase has often been suggested to drive wound angiogenesis, so it was possible that an upregulated inflammatory response could have created the increase in capillary content and vascular permeability that was seen in the wounds of PEDF^−/−^ mice. However, our results show that the loss of PEDF has only a small effect on the inflammatory phase of wound healing. These findings uncouple inflammation and the regulation of angiogenesis and vessel permeability in wounds. Therefore, the mechanisms that control inflammation and capillary permeability in wounds appear to be primarily independent.

The data presented herein reveal that PEDF is principally a wound resolution factor, and demonstrate PEDF’s functional role in wound healing. Together with our previous studies, these results indicate that loss of PEDF in wounds affects many processes, but primarily angiogenesis. These studies have advanced our understanding of the role of PEDF in wound healing, and suggest that PEDF might be a novel therapeutics that would encourage wound resolution, decrease scar formation and promote tissue regeneration. The findings may also be important to the many other pathological conditions that involve exuberant angiogenesis and/or fibrosis, such as malignancies and fibrotic diseases. In this context, PEDF is already under investigation as a potential therapeutic for cancer, a situation in which the anti-angiogenic function of PEDF could be exploited^44^. For fibrotic diseases, however, additional information on the specific pathways by which PEDF may regulate angiogenesis versus collagen synthesis will be needed to fully realize any therapeutic approach.

## Electronic supplementary material

Supplementary Fig. 1

Fig 1 Supplementary Dataset

Fig 2 Supplementary Dataset

Fig 3 Supplementary Dataset

Fig 4 Supplementary Dataset

Fig 5 Supplementary Dataset

Fig 6 Supplementary Dataset

Fig 7 Supplementary Dataset

Suppl Fig 1 Supplementary Dataset
