# Supplementary material for: Pigment Epithelium-Derived Factor (PEDF) as a Regulator of Wound Angiogenesis
Source: Sci Rep. 2018 Jul 24;8:11142. doi: 10.1038/s41598-018-29465-9 (PMC6057962; doi:10.1038/s41598-018-29465-9)
Supplement: Supplementary file 1 — Supplementary Fig. 1 [file 41598_2018_29465_MOESM1_ESM.pdf]

Pigment Epithelium-Derived Factor (PEDF) as a Regulator of Wound Angiogenesis

Elizabeth R. Michalczyk[1,3]; Lin Chen[1,3]; David Fine[1]; Yan Zhao[1]; Emman Mascarinas[2]; Paul J. Grippo[2]; Luisa A. DiPietro[1*]

1. Center for Wound Healing and Tissue Regeneration, College of Dentistry, University of Illinois at Chicago, Chicago, IL, USA

2. Division of Gastroenterology and Hepatology, Department of Medicine, College of Medicine at Chicago, University of Illinois at Chicago, Chicago, IL, USA

3. These authors contributed equally to this work.

***Correspondence:**

Luisa A. DiPietro

Center for Wound Healing & Tissue Regeneration (MC 859)

University of Illinois at Chicago

801 S. Paulina Street

Chicago, IL 60612-7211

Phone: 312.355.0432

Fax: 312.996.0943

E-mail: Ldipiet@uic.edu

Suppl Figure 1

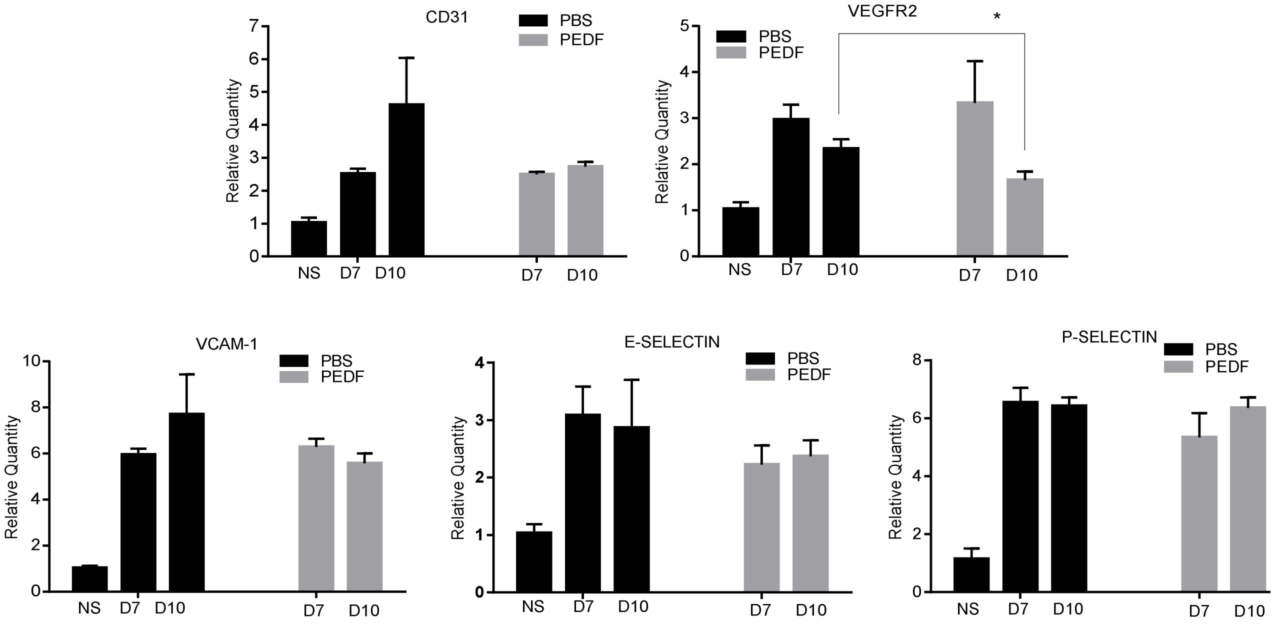

Suppl. Figure 1. PEDF regulation of the expression of endothelial cell markers and receptors in skin wounds. Three mm excisional wounds were treated with PEDF topically. mRNA expression of CD31, VEGFR2, VCAM-1, E-Selectin, and P-Selectin in wounds of days 7 and 10 was examined by semi-quantitative real time PCR. The average value for normal skin was set at 1. Mean±SEM; n=5.*$p<0.05$, Student's t-tests was used for statistical analysis.
